# A scoping review of effects of acute exercise on executive function: evidence from event-related potentials

**DOI:** 10.3389/fpsyg.2025.1599861

**Published:** 2025-05-01

**Authors:** Zhidong Cai, Lin Shi, Wenjie Wu, Liang Meng, Yao Ru, Moulin Wu

**Affiliations:** ^1^Department of Physical Education, Suzhou University of Science and Technology, Suzhou, China; ^2^School of Physical Education, Chengdu Sports University, Chengdu, China

**Keywords:** cognition, executive function, acute exercise, event-related potential, EEG

## Abstract

**Background:**

Although the acute effects of exercise on executive function are extensively documented in the field of exercise psychology, a thorough assessment of neuroelectric brain activity that is underlying executive function following acute exercise is absent. This systematic review investigated the effects of acute exercise on event-related potentials through their amplitude and latency.

**Methods:**

Six electronic databases were searched from their inception to April 15, 2024. This review analyzed the influence of variables such as exercise dosage, test duration, population characteristics, and physical fitness on event-related potential components and executive function. The proportions of positive and null or negative effects across all studies were systematically assessed.

**Results:**

In total, 52 studies were included in the analysis. The results revealed that 45 (86.5%) of the 52 studies focused on inhibitory control, with moderate-intensity aerobic exercise lasting 16–35 min demonstrating a positive effect on event-related potential components. Nine event-related potential components were examined, with P3 (observed in 43 studies), N2 (17 studies), and N1 (5 studies) being the most frequently reported. Thirty-seven studies (86%) demonstrated that exercise enhanced P3 wave amplitude, whereas 13 studies (27.1%) observed a reduction in latency. Eight studies (53.3%) indicated an increase in N2 wave amplitude following exercise, whereas two studies (13.3%) reported a decrease in latency.

**Conclusion:**

Moderate-intensity acute aerobic exercise lasting 16–35 min demonstrates a positive impact on both executive function performance and event-related potential components, with effects lasting approximately 30 min. The optimal intervention dosage for resistance exercise, mind–body exercise, and other types of exercise necessitates further investigation in future studies.

## Introduction

Maintaining optimal cognitive performance is essential for promoting human well-being. In recent decades, there has been substantial interest in the correlation between cognitive function and physical exercise. Engaging in an active lifestyle or participating in regular physical exercise is correlated with enhanced brain health (Erickson et al., 2019). Research indicates that both acute and chronic exercise can significantly enhance cognitive function, mitigate cognitive decline in the elderly, and positively influence academic performance among children and adolescents. Chronic and acute exercise have distinct influences on cognitive functions and neurophysiological responses. Chronic exercise, such as regular aerobic training, enhance executive function through sustained neuroplasticity (Aly and Kojima, 2020; Kamijo and Takeda, 2009). In contrast, acute exercise focuses on transient neurophysiological changes, such as increased arousal and neurotransmitter availability. This review specifically examines the acute effects of exercise on executive function, as measured by ERP components.

Executive function is a higher-order cognitive process characterized by active regulation of non-automatic behaviors to achieve consciously set goals (Diamond, 2013). Core executive function consists of three distinguishable subdomains: inhibitory control, working memory, and cognitive flexibility (Diamond, 2020). Specifically, inhibitory control refers to the ability to actively suppress dominant stimuli or interfering information and filter out irrelevant cues (Ren et al., 2021); working memory is defined as the capacity to temporarily maintain and manipulate relevant information (Miyake et al., 2000); and cognitive flexibility is the ability to flexibly shift between mental frameworks, operations, or conceptual representations (Kofler et al., 2019). In 2003, Colcombe et al. demonstrated that the effects of exercise on cognitive function are selective, particularly with respect to executive function (Colcombe and Kramer, 2003). Since then, the enhancement of executive function through exercise has emerged as a significant area of research. Historically, the field has primarily relied on behavioral performance metrics (e.g., response accuracy and reaction time) as indicators of cognitive function. While these metrics assess whether exercise is cognitively beneficial, they are less effective at detecting subtle changes in cognitive processes. In recent years, advanced neuroscientific techniques, including functional near-infrared spectroscopy (Cai et al., 2023), functional magnetic resonance imaging (fMRI) (Yin et al., 2022), and event-related potentials (ERP) (Zhao et al., 2022), have been extensively utilized to investigate the relationship between exercise and cognitive functions. Among these techniques, ERP offers high temporal resolution and yields insights into covert cognitive processes that occur between stimulus presentation and response execution. It is particularly effective for elucidating the neurobiological mechanisms underlying motor induction that may not be apparent from behavioral performance alone. As cognitive neuroscience has advanced, an increasing number of studies have utilized ERP to examine how acute exercise enhances executive function, thereby addressing the limitations of behavioral indicators. Chang et al., demonstrated that 20 min of power cycling resulted in reduced Stroop reaction times, increased P3 amplitude, and decreased N450 amplitude (Chang et al., 2017). Bae et al. reported that after 30 min of running, the P3 amplitude at Pz was significantly greater and the latency was notably shorter under exercise conditions compared to control conditions (Bae and Masaki, 2019).

Numerous ERP components have been investigated in studies examining the effects of acute exercise on executive function. Commonly utilized components include P3, N2, and N450, which are employed in research on various aspects of executive function, including inhibition, working memory, and cognitive flexibility. Although previous reviews have focused exclusively on P3 (Kao et al., 2020a,b; Kao et al., 2022a; Pedroso et al., 2017), other ERP components remain inadequately summarized and lack a cohesive understanding. The primary objective of this study was to systematically review the immediate effects of acute exercise on neurophysiological representations of executive function. Secondary objectives were to investigate the moderating effects of exercise intensity, duration, timing of assessment, and individual differences.

## Methods

### Search strategy

This systematic review was conducted following the guidelines of the Preferred Reporting Items for Systematic Reviews and Meta-Analyses extension for scoping review (PRISMA-ScR) (Tricco et al., 2018). The literature search strategy was constructed based on the PICO framework (Population, Intervention, Comparison, Outcome) to systematically identify eligible studies. In this study, P refers to humans, I refers to acute exercise intervention, C refers to rest, reading or another kind of exercise, O refers to ERP components, behavioral indices of executive functions. We searched six electronic databases (PubMed, Cochrane Library, Web of Science, Scopus, Wangfang, and the National Knowledge Infrastructure of China) from inception to October 15, 2024. To ensure a thorough exploration, we also reviewed the reference lists of systematic reviews published in the previous 3 years and conducted a manual search for additional relevant studies. The research strategy implemented in this review is described as follows:

#1 acute exercise OR a single bout exercise OR physical exercise OR physical activity OR aerobic exercise OR resistance exercise OR strength exercise OR stretching OR mind-body exercise OR flexibility exercise OR coordinative training OR multicomponent exercise

#2 cognition OR executive function OR executive control

#3 event-related potentials OR ERP OR electroencephalography

#4 #1 AND #2 AND #3

### Inclusion and exclusion criteria

The inclusion criteria for this study consisted of three key aspects: (1) the study must be an experimental investigation focusing on acute exercise interventions for cognitive function; and (2) the outcome measures must include both executive function and ERP components.

The exclusion criteria comprised two main aspects: (1) the intervention protocol included confounding factors associated with non-exercise interventions; (2) the study type included meta-analyses, reviews, case reports, conference papers, commentaries, and other non-original research articles; and (3) data could not be extracted from the studies, even after contacting the authors.

### Risk of bias assessment

Two independent reviewers (S.L. and W.M.L.) evaluated the quality of the selected studies. Both reviewers had previously received training in evaluating the risk of bias in research studies. Initially, there was strong agreement among the reviewers in assigning risk of bias scores, resulting in a concordance rate of 85.8%. Discrepancies between the reviewers were addressed through a consensus meeting. The methodological quality of the studies was assessed using an adapted version of the PEDro checklist, as previously employed by researchers (Kao et al., 2022b).

The criteria considered in this review included: (1) random allocation; (2) concealed allocation; (3) comparability at baseline; (4) blinded assessment; (5) between-group comparison; (6) outcome point estimates; (7) exercise specifications; and (8) evaluation of ERP components. Reviewers awarded a score of 0 if a study failed to meet a criterion or if the information was unclear, and a score of 1 if the criterion was met. Studies that utilized within-subject designs received a score of 1 for the criteria of random allocation, concealed allocation, baseline comparability, and between-group comparison.

### Study selection process

Two reviewers were tasked with screening the articles. First, titles and abstracts were examined to identify studies that met the inclusion criteria. Second, the full text of the selected studies was reviewed to determine final inclusion. Disagreements between reviewers were resolved through meetings involving both reviewers and field experts.

### Data collection and extraction process

The final studies included in the review were rigorously examined, and the following data were extracted and recorded in the database: (a) the name of the first author and the publication year; (b) sample size, age, sex, and other relevant characteristics of the study population; (c) experimental protocol; (d) ERP components and tasks utilized; and (e) the primary findings.

### Data synthesis

The descriptive information for all included studies was summarized in Table 1. In cases where meta-analyses were not feasible or suitable, the effects of the studies were summarized based on the recommendation to synthesize and present the findings of the review (Mckenzie and Brennan, 2021). The proportions of positive and null/negative effects across all studies were calculated.

**Table 1:** Basic characteristics of the literature included in the study.

Study ID	Participant	Experimental protocol	ERP components, electrode position	Cognitive domain	Main findings
Aly (2020)	Young adults Age: 23 N:40	Randomized control design T:20 min of cycling I: moderate, 67.5% HR_max_ D: NR C:20 min of resting	P2, N2c, P3 Fz, Pz	Inhibition: Flanker task	Acute moderate-intensity exercise facilitated response times and increased the P2, N2c, and P3 amplitudes.
Akatsuka et al. (2015)	Young adult Age:19.8 N:10	Cross-over design T:15 min of treadmill running I: moderate, 50% VO2max D:5 min C: 20 min of rest	N140 Fz, Cz	Inhibition: Go/no-go task	N140 amplitude at Fz and Cz was significantly increased in the experimental group under no-go conditions, while no significant differences were observed in the control group.
Bae and Masaki (2019)	Young adults age:21.4 N:29	Cross-over design T:30 min of treadmill running I: moderate, 70% HRmax D:20-30 min C: 30 min of reading	P3 Pz	Switching: Task-switching	Compared to the control condition, P3 amplitude was larger and latency shorter at Pz under exercise conditions.
Bailey et al. (2021)	Young adults age:22.5 N:210	Cross-over design T:40 min of treadmill running I: light: 35%VO2_max_; high, 70%VO2_max_ D: 20-25 min C: 40 min of video	P3, N2 FCz, Cz	Inhibition: Go/No-go Flanker	Faster response times (RT) and improved accuracy following 70% VO2 max exercise compared to rest, but not 35% VO2 max; RTs and accuracy did not differ between 35% VO2 max exercise and rest conditions. N2 and P3 amplitudes were larger following 70% VO2max exercise for the food-based go/no-go task compared to rest and 35% VO2max exercise
Chacko et al. (2020)	Young adults age:26.8 N:15	Cross-over design T:30 min of treadmill running I: high, 67.5% HRR D: immediately C: 30 min of reading	N1, P3 PO7, PO8, Cz, CPz	Inhibition: Go/No-go	Vigorous-intensity aerobic exercise did not affect executive function but had significant effects on the reactive functions related to selective attention in parietal areas (indexed by the N1 amplitude) and perceptual awareness in the anterior insula
Chang et al. (2017)	Young adults age:22.6 N:30	Cross-over design T:30 min of cycling I: moderate, 60-70%HRR D:15 min C: 30 min of reading	N1, N2, P3, N450 Fz, Cz, Pz	Inhibition: Stroop	Increased P3 amplitude, decreased N450 amplitude, and shorter N450 latency were observed, with no effects on N1 and N2 components.
Chu et al. (2015)	Young adults age:21.5 N:21	Cross-over design T: 30 min of treadmill running I: moderate, 65–75% HRmax D:10 min C: 30 min of reading	P3, N1 Fz, Cz, Pz	Inhibition: Stop-signal	Acute exercise shortened No-go reaction times compared to the control group, with no change in Go reaction times; P3 amplitude and latency improved, while N1 remained unchanged.
Chu et al. (2017)	Preadolescent children/young adults age:10.5/20.4 N:20/20	Cross-over design T:30 min of treadmill running I: moderate, 65-75%HRR D: Immediate C: 30 min of reading	P3, conflict SP Fz, Cz, Pz, CP3, P3, CP4, P4	Inhibition: Stroop	P3 amplitude was larger and conflict SP amplitude was smaller in the exercise condition compared to the control condition. Larger P3 amplitude led to reduced conflict SP amplitude in preadolescent children.
Dimitrova et al. (2017)	Young adults/older adults Age:23.2/70.7 N:58	Cross-over design T: 20 min exercise game (cycling, screen game) I: moderate, 60%HRR D:20 min C: cycling	N2, P3 Cz	Inhibition: Flanker	Increased amplitude of N2 and P3 for both groups; elderly showed greater increase under incongruent conditions.
Drollette et al. (2014)	Children Age: 9.7 N: 40人	Cross-over design T: 20 min of treadmill walking I: moderate, 60–70% HRmax D:22.5 min C: 20 min of rest	P3, N2 Fz, FCz, Oz	Inhibition: Flanker	Higher performers maintained accuracy and exhibited no change in P3 amplitude compared to seated rest. Lower performers increased P3 amplitude following exercise. Both groups displayed smaller N2 amplitude and shorter P3 latency following exercise.
Du Rietz et al. (2019)	Young adults Age: 21.5 N:26 (male)	Cross-over design T:30 min of cycling I: high, HR,190, RPE19 D:30 min C:30 min of rest	P3, N2, CNV Cz	Inhibition: Flanker, Fast task, CPT-OX	Increased Go P3 amplitude; no effect on CNV, Cue P3, No-go P3. Increased Delta power post-exercise; no effect on cognitive performance.
Hillman et al. (2003)	Young adults Age:20.5 N:20	Cross-over design T: 30 min of treadmill running I: high, 83.5% HRmax D:48 min C:30 min of rest	P3 Fz, Cz, Pz, Oz	Inhibition: Flanker	Increased P3 amplitude after acute exercise. Acute exercise enhances executive function by increasing neural resource allocation cognitive processing and categorization speed.
Hillman et al. (2009)	Children Age:9.5 N:20	Cross-over design T: 20 min of treadmill walking I: moderate, 60% HRmax D:25.4 min C:20 min of rest	P3 Fz, FCz, Cz, CPz, Pz	Inhibition: Flanker	Increased P3 amplitude, and response accuracy latency. Acute aerobic exercise can improve cognitive control and academic performance in children.
Hsieh et al. (2018)	Older adults/ young adults Age:24/70 N:24/20 (male)	Cross-over design T: 30 min of treadmill running I: moderate, 60–70% HRR D:15 min C:30 min of video	P3, N450 FCz, Cz, CPz, Pz	Inhibition: Stroop	Acute exercise resulted in shorter response time, larger P3 amplitude, and smaller N450 amplitude regardless of age or congruency.
Hung et al. (2016)	ADHD Age:10 N:34	Cross-over design T: 20 min of treadmill running I: moderate, 50-70%HRR D: NR C: video	P3 Fz, Cz, Pz	Switching: Task switching	Under mixed-task conditions, P3 amplitude increased post-exercise, showing reduced overall task-switching costs. Single bouts of moderate-intensity aerobic exercise may positively impact task-switching in children with ADHD.
Kamijo et al. (2004a)	Young adults Age:22–33 N: 12	Cross-over design T:18 min of cycling I:HR, low, 84.43 bpm; moderate,118.17 bpm; high 190.17 bpm D:3 min C: rest	CNV, P3 Fz, Cz, Pz	Inhibition: Go-no go	Moderate-intensity exercise increased P3 amplitude, while high-intensity exercise decreased it. Differences in exercise intensity influenced information processing in the central nervous system.
Kamijo et al. (2007)	Young adults Age:25.7 N: 12	Cross-over design T:20 min cycling I: low, RPE = 11, moderate, RPE = 13, high, RPE = 15 D: Immediate C: rest	P3 Fz, C3, C4, Cz	Inhibition: Flanker	Moderate and low intensities increased P3 amplitude, while high intensity showed no significant change. The latency of P3 was shortened under inconsistent conditions, and reaction times were shortened across all exercise conditions.
Kamijo et al. (2009)	Older adults/young adults Age:56.5/21.8 N:12/12	Cross-over design T:25 min of cycling I: low 30% VO2max, moderate:50% VO2max D: Immediate C: rest	P3 Fz, Cz, Pz	Inhibition: Flanker	Moderate intensity increased P3 amplitude in young adults; low and moderate intensities shortened P3 latency in both young and older adults.
Kao et al. (2017)	Young adults Age:19.2 N:64	Cross-over design T:16 min of continuous aerobic exercise running/9 min of high-intensity interval running I: moderate, 60–70% HRmax /high,90% HRmax D:20 min C: 20 min of rest	P3 Fz, Cz, Pz	Inhibition: Flanker	Compared to the control condition, both exercise conditions shortened reaction times, with HIIE improving accuracy. Compared to the control, P3 amplitude was significantly increased after aerobic exercise, while HIIE reduced amplitude and latency. The two exercise types differ in their effects on inhibition, with HIIE potentially being a more efficient method for enhancing cognition.
Kao et al. (2020a,b)	Young adults Age:19.2 N:23	Cross-over design T: 20 min of treadmill running I: moderate, 60–70% HRmax D:30 min C:20 min of rest	P3, ERD Cz, Pz, CPz	Working memory: N-back	After AE, target stimulus P3 amplitude increased, and frontal α desynchronization was stronger in the 2-back compared with the 1-back task following AE. AE may temporarily enhance working memory.
Kao et al. (2018)	Young adults Age:21.5 N:36	Randomized control design T: 20 min of treadmill running I: moderate, 70% HRmax; high, 88.6 HRmax D:30 min C:20 min of rest	P3 Fz, FCz, Cz, CPz	Inhibition: Flanker task	MICE and HIIT resulted in shorter reaction times compared to the rest. A larger P3 amplitude was observed following MICE compared to HIIT and rest, whereas HIIT resulted in shorter P3 latency compared to rest. MICE and HIIT have similar short-term facilitatory effects on inhibitory control, which may have different effects on the neuroelectrical mechanisms.
Ligeza et al. (2018)	Young adults Age:24.9 N:18 (male)	Cross-over design T: 24 min of treadmill running I: moderate, 64%HR_max_; high,83% HR_max_ D: 10 min C:24 min of reading	P3b, N2 Fz, FCz, Cz, Pz	Inhibition: Flanker	Moderate-intensity exercise significantly increased N2 amplitude compared to both sedentary and high-intensity interval exercise. No significant differences in P3 amplitude were observed.
Lind et al. (2019)	Children Age:11.8 N:81	Randomized control design T:20SRF/SWF I: moderate, 60-80%HRR; high,70-100%HRR D:20 min C:20 min of video	P3 Fz, Cz, POz	Inhibition: Flanker	SRF improved inhibitory control more than SWF. At Fz, P3 amplitude under consistent conditions was higher in SRF than RF and higher in inconsistent conditions than SWF.
Ludyga et al. (2017)	ADHD/healthy children Age:12.8/13.5 N:16/17	Cross-over design T:20 min of cycling /coordinative exercise I:moderate:65-70%HRmax D:10 min C:20 min of video	P3 P1, P2, P3, P4, Pz	Inhibition: Flanker	For ADHD, compared to control conditions, both exercise groups had increased P3 amplitude and reduced reaction times; aerobic exercise was more effective than coordination exercise.
McGowan et al. (2019)	Young adults Age:19.2 N: 58	Cross-over design T:20 min of running /coordinative exercise I:moderate:71.3%HRmax D:10 min C:20 min of walking	P3 C1/Z/2, CP1/Z/2, P1/Z/2	Inhibition: Flanker	Enhancements in both reaction time and neuroelectric indices of attention were observed in response to the exercise condition.
O'Leary et al. (2011)	Young adults Age:21.2 N:36	Cross-over design T:20 min of exergame/cycling/ videogame I: moderate: 60% VO2max D:22.2 min C:20 min of rest	P3 Fz, FCz, Cz, CPz, Pz	Inhibition: Flanker	Aerobic exercise increased P3 amplitude and shortened reaction times compared to rest. However, exergaming and seated videogames did not provide similar benefits.
Pontifex et al. (2013)	ADHD/healthy children Age:9.5/9.8 N:20/20	Cross-over design T: 20 min of treadmill running I:moderate:65-75%HRmax D:16-27 min C:20 min of reading	P3, ERN Fz, FCz, Cz, CPz, Pz, POz, Oz	Inhibition: Flanker	Post-exercise, P3 amplitude increased, latency shortened, and ERN amplitude increased in both conditions.
Pontifex and Hillman (2007)	Young adults Age:20.2 N:41	Cross-over design T:11.8 min cycling I:moderate:60%HRmax D: during acute cycling C: resting	N1, N2, P2, P3 Fz, FCz, Cz, CPz, Pz	Inhibition: Flanker	Under exercise conditions, N1 amplitude decreased at parietal, N2 amplitude decreased globally, and P2 and P3 increased in the frontal region. N2 and P3 latency were prolonged.
Pontifex et al. (2021)	Yang adults Age:19.5 N:70	Cross-over design T:20 min cycling I:moderate:70.4%HRmax D: 5 min C: 20 min of video	P3, ERN Fz, FCz, Cz, CPz, Pz	Inhibition: Flanker	Both high anxious and low-anxious individuals exhibited faster and more accurate responses following 20 min of moderate-intensity aerobic exercise. Post-exercise, P3 amplitude increased, latency shortened, and ERN amplitude increased in both groups.
Scudder et al. (2012)	Young adults Age:19.7 N:13	Cross-over design T: 30 min of treadmill running I:moderate:60%HRmax D:23.5 min C:30 min of reading	P3, N2 Fz, FCz, Cz, CPz, Pz	Inhibition: AX-continuous performance	P3 amplitude increased at midline-parietal sites for both target trials and non-target trials. Acute aerobic exercise may facilitate goal maintenance processes and enable individuals to better inhibit extraneous neural activity.
Stroth et al. (2009)	Adolescents Age:14.2 N:35	Cross-over design T:20 min of running I:moderate:60%HRmax D: NR C:20 min of resting	CNV, N2, P3 FC3, FC4, C3, C4, O1, O2	Inhibition: Flanker, Go-no go	High fitness levels showed larger CNV amplitudes and smaller N2 amplitudes. P3 amplitude was not related to acute exercise or fitness levels. Physical fitness, but not an acute aerobic exercise enhances cognitive processing by increasing attentional allocation to stimulus encoding during task preparation.
Swatridge et al. (2017)	Older adults (chronic stroke) Age:57.8 N:9	Cross-over design T:22 min of walking I:moderate:45-50%HRR D: immediate, 20 min,40 min C:20 min of resting	P3 Fz	Inhibition: Flanker	P300 amplitude at Fz was greater 40 min after exercise compared with after rest. P300 latency was also shorter at 20 min after exercise compared with after rest. Differences in behavioral performance after exercise were not significant.
Themanson and Hillman (2006)	Young adults Age:20.1 N:20	Cross-over design T: 20 min of treadmill running I:moderate:60%HRmax D:48 min C:30 min of reading	N2, ERN, Pe Fz, Cz, Pz	Inhibition: Flanker	High fitness levels showed larger ERN and Pe amplitudes with no relation to acute exercise. Cardiorespiratory fitness, but not acute aerobic exercise, may be beneficial to behavioral and neuroelectric indices.
Torbeyns et al. (2016)	Young adults Age:35 N:23	Cross-over design T: 30 min of cycling I: low, RPE3.3 D: during acute cycling C: 30 min of resting	N2, P3, N450 Fp1, Fp2, F4, F3, Fz, F7, F8, FC1, FC2, P3, P4, Pz, P7, P8, PO9, PO10	Inhibition: Stroop	No differences in accuracy between conditions; reaction times were significantly shorter under exercise conditions compared to sedentary. No differences in N2 or P3 amplitudes or latencies.
Tsai et al. (2018)	MCI Age:65 N:66	Randomized control design T:30 min of cycling/ resistance exercise I: moderate, 65–75%/ HRR, 75% 1RM D:5 min C:30 min of reading	P3 Fz, Cz, Pz	Inhibition: Flanker	AE and RE improved behavioral performance and increased ERP P3 amplitude in individuals with aMCI.
Tsai et al. (2014)	Young adults (male) Age:22.9 N:60	Randomized control design T:40 min resistance exercise I: moderate, 50%1RM; high, 80% 1RM D:5 min C:45 min of reading	P3 Fz, Cz, Pz	Inhibition: Go/No-Go	The acute RE could not only benefit the subjects’ behavioral (i.e., RTs and accuracy) performance but also increase the P3 amplitude.
Tsai et al. (2016)	Young adults (male) Age:22.5 N:60	Non-randomized between-subject design T:36 min of running I: moderate, 50%1RM; high, 80% 1RM D:5 min C:47 min of reading	P3 Pz	Switching: Task-switching	Acute AE decreased reaction times, only the EI_H_ group showed a smaller switching cost and larger P3 amplitudes.
Tsai et al. (2021)	Older adults Age: 60.6 N:21	Cross-over design T: 30 min of resistance exercise I:moderate: 52.5%HRR; high: 72.5% D:5 min C: 30 min of reading	P3 Pz, Cz, CPz	Working memory: Delayed matching task	ARs were significantly increased only via the MICE intervention mode, the acute HIIT and MICE interventions improved RT performance and increased ERP P3 amplitudes in the late middle-aged and older adults under consideration.
Vonk et al. (2019)	Young adults Age:23.4 N:22	Cross-over design T: 30 min of resistance exercise I:moderate: 70%10RM D:10/20/30/40 min C: loadless movement	P3 Pz	Inhibition: Stroop	40 min post-intervention, P3 amplitude increased in both congruent and incongruent conditions. Reaction times in congruent and incongruent conditions were shorter compared to pre-exercise.
Yu et al. (2022)	Young adults (criminal) Age:30.1 N:15	Cross-over design T:30 min of cycling I:60%HRR D:5 min C:30 min of reading	P3, N2, ERN, Pe Pz, PCz	Inhibition: emotional stop signal task	Acute aerobic exercise significantly shortened stop-signal reaction times compared to control, but no significant differences in ERP components.
Wang et al. (2016)	Young adults (MA) Age:33 N:92	Randomized control design T:30 min of cycling I: moderate,65–75%/ HRmax D:20 min C:30 min of reading	N2 Fz, Cz, Pz	Inhibition: Go-no go	Moderate-intensity exercise resulted in shorter go task reaction times and increased no-go accuracy, with larger no-go N2 amplitudes. MA craving scores were lower than reading control sessions during, immediately following, and 50 min after the exercise session.
Wang et al. (2015)	Young adults (MA) Age: 31.5 N: 24	Cross-over design T:30 min of cycling /resistance exercise I:high:81.3% HRmax D: NR C:30 min of reading	N2, P3 Fz, Cz, Pz	Inhibition: Go-no go	Acute exercise facilitated inhibitory performance in both the standard and MA-related Go/Nogo tasks. A larger N2 amplitude, but not a larger P3 amplitude was observed during both tasks in the exercise session and the Nogo condition compared with the reading control session and the Go condition.
Wu et al. (2019)	Young adults Age:21 N:30	Cross-over design T:30 min of cycling /resistance exercise I:moderate: 60-70%HRR, 70%10RM D:30 min C:20 min of reading	P3b, N1 Fz, Cz, Pz	Switching: Task-switching	Both AE and RE elicited shorter response times in global switching and local switching compared to control. Larger P3b amplitudes (but not N1 amplitudes) were evident in global switching and local switching, regardless of exercise modality.
Winneke et al. (2019)	Young adults Age:25.6 N:11	Cross-over design T:20 min of cycling I: moderate,60% VO2max D:2.56 min C:20 min of reading	P3, N2 Fz	Inhibition: Flanker	No effect of exercise was found for behavioral data. P3 latencies were shorter following exercise as compared to rest. The N2 amplitude data suggest that exercise seems to prevent a decline in resources of attentional control over time.
Xie et al. (2020)	Young adults (obesity) Age:24.5 N:16 (male)	Cross-over design T:20 min of cycling (HIIE) I: high,80-90%HRmax D:15 min C:20 min of reading	P3, LPP Fz	Inhibition: Flanker	HIIE increased LPP amplitude, and shortened response time, but did not affect P3 amplitude. Acute HIIE has a generally beneficial effect on basic information processing and inhibitory control among young adult males with obesity.
Li, G. G. (2020)	Young adults 30 (female)	Randomized control design T:30 min of exergame I: moderate: 60-70%HRmax D:15-20 min C: 30 min of cycling	P2, N2, P3b Fz, FCz, C4, CP3, P4	Inhibition: Flanker	The exergames had shorter P2, N2, and P3b latencies compared to the aerobic exercise group. Exergames can improve the individual’s ability to inhibit and control, as well as aerobic exercise is an effective cognitive training method.
Li et al. (2020)	Young adults Age:22 N:12	Cross-over design T:40 min of Tai Chi I: moderate: 60-69%HRmax D: NR C: rest	N2, P3 F3, Fz, F4, P3, Pz, P4	Inhibition: Flanker	Paired t-tests showed shorter reaction times under both congruent and incongruent conditions. N2 and P3 amplitudes increased. Tai Chi can improve the inhibition function of college students. The neural mechanism shows a significant increase in N2 amplitude in the prefrontal lobe and a significant increase in P3 amplitude in the parietal occipital lobe.
Wang and Zhou (2014)	Young adults Age:20.7 N:100	Randomized control design T:20 min of cycling I: low,45–50%/moderate,65–70%/high,80-95%HRmax D: immediately C:20 min of reading	N2, P3 Fz, Cz, Pz	Inhibition: Go-no go	The Go N2 amplitude in the moderate-intensity group was significantly lower than in the control and high-intensity groups. The Go N2 latency in the high-intensity group was significantly shorter than in the control and low-intensity groups. No differences in No-Go N2 amplitude or latency between groups. The Go P3 and No-Go P3 amplitudes in the moderate-intensity group were significantly higher than in the control and low-intensity groups. The Go and No-Go P3 amplitudes in the high-intensity group were significantly higher than in the control group, with no differences in P3 latency.
Wen and Tsai, 2020	Young adults Age:33 N:32	Randomized control design T:35 min of dance and dumbbell exercise I: moderate,60% HRR D: 28.4 min C:25 min of reading	N2, P2, P3 Fz, Cz	Inhibition: Flanker	Exercise shortened the N2 and P3 latencies, and increased the N2, P3 amplitudes. A combination of acute moderate-intensity aerobic and resistance exercise may improve the neurophysiological inhibitory control performance of obese women.
Won et al. (2019)	Young adults Age:24.9 N:2	Cross-over design T:30 min of running/ futsal I: moderate,67.5%/68.2% HR_max_ D: NR C:30 min of rest	P3 Fz, Cz, Pz	Inhibition: Stroop	Both exercises shortened the reaction time of the Stroop and increased the amplitude of the P3 compared to the control group.
Zhou and Qin (2019)	Young adults Age:20.1 N: 72	Randomized control design T:25 min of cycling I: moderate,66.5 HRmax D: 15 min C:25 min of reading	P2, N2, P3b, N450 Fz, Cz, Pz	Inhibition: Stroop	Acute exercise increased P2 amplitudes and did not influence accuracy or response time. Acute moderate-intensity exercise may have a generally beneficial effect on the mobilization of attentional resources and exercise-related physiological arousal.
Zhu and Zhou (2016)	Young adults Age:20.7 N:17	Cross-over design T:30 min of cycling I:moderate:60–69HRmax D: NR C:30 min of reading	N2, P3 Fz, FCz, Cz, CPz	Inhibition: Go-no go	N2 amplitude was significantly lower under exercise conditions compared to control conditions, while P3 amplitude was significantly higher under exercise conditions. Moderate-intensity aerobic exercises improved inhibition. It may be due to the more rapid recognition of inhibition signal, and faster distribution of cognitive resources.

ADHD, attention-deficit/hyperactivity disorder; AE, aerobic exercise; AR, Accuracy rate; C, control group; CPT-OX, cued continuous performance task; D, duration; EI_H_, higher fitness group; ERN, error-related negativity; HIIE, high intensity interval exercise; HR, heart rate; HRmax, maximum heart rate; HRR, heart rate reserve; I, intensity; LPP, late positive potential; MCI, mild cognitive impairment; MA, methamphetamine addict; MICE, moderate intensity continuous exercise; N, sample size; NR, no report; RE, resistance exercise; RF, resting; RM, repetition maximum; RPE, rating of perceived exertion; RT, reaction time; SP, sustained potential; SRF, small-sided real football game; SWF, small-sided walking football games; VO2max, maximum oxygen consumption.

## Event-related potential

ERP refers to brain potentials recorded from the scalp surface, obtained by averaging responses to specific stimuli during cognitive processing. ERP reflects neurophysiological changes occurring in the brain during cognitive tasks, thus the term “cognitive potential.” Walter discovered the first cognitively relevant ERP component, the Contingent Negative Variation (CNV), which marked the beginning of modern ERP research. The discovery of the P300 (P3) component by Sutton et al. (1965) established ERP as a significant topic in cognitive psychology. ERP is a non-invasive technique that offers high temporal resolution at the millisecond level, facilitating a detailed investigation of the “black box” of brain function and enabling the assessment of complex cognitive processes. An increasing number of studies have employed ERP techniques to investigate the underlying mechanisms linking acute exercise to cognitive function.

An ERP component is a neural signal that originates from a specific neuroanatomical region when the brain performs a particular computational operation and can be recorded from the scalp. Determining the nature of the psychological or neural processes reflected by an ERP component is a central issue in ERP research. Traditionally, ERP components are classified based on their polarity and latency. For instance, the P300 component reflects a positive wave observed at approximately 300 ms. Classical ERP components include P1, N1, P2, N2, and P3, with P1, N1, and P2 being exogenous components influenced by the stimulus, while N2 and P3 are endogenous components related to the individual’s cognitive state (Zhao, 2007). Consequently, P3 and N2 are the most frequently utilized components in studying executive function within the context of acute exercise interventions (Table 2).

**Table 2:** Summary of event-related potential components: latency, associated brain regions, and cognitive functions.

Component	Latency (ms)	Brain regions	Cognitive functions
N1	50–150	Temporal lobe, Parietal lobe	Early sensory processing
P2	150–250	Prefrontal cortex, Central regions	Stimulus categorization, initial semantic processing
N2	200–350	Anterior cingulate cortex, Prefrontal cortex	Conflict monitoring, error detection
P3	300–600	Parietal lobe, Central regions	Decision-making, working memory, attention resource allocation
CNV	500–1500 (S1-S2 interval)	Prefrontal cortex, Anterior cingulate cortex, Motor cortex	Anticipation/behavioral preparation, maintenance of attentional resources, temporal interval estimation, motor preparation/inhibition

The P3 component is a key ERP component for studying executive function in acute exercise interventions and is subdivided into P3b and P3a. P3b was first identified by Sutton during Oddball experiments involving rare stimuli, typically recorded as a positive deflection occurring between 300 and 800 ms (Sutton et al., 1965). P3b has been observed in both the frontal and parietal lobes, with the maximum amplitude recorded in the parietal lobe. Although the precise interpretation of P3 remains controversial, P3b is often associated with situational updating, stimulus evaluation, and resource allocation (Coles and Rugg, 1995). Halgren et al. found that P3-like signals can also be recorded from the hippocampal structures and the amygdala (Halgren et al., 1980). The hypothesis that the hippocampus is one of the sources of P3 is widely accepted among scholars. P3a is a component of P3 elicited by novel, contingent stimuli, with the maximum amplitude observed in the posterior frontal lobe, significantly more anterior than that of P3b.

The N2 component is characterized by a maximum negative deflection occurring in the frontal-central region approximately 150 to 350 ms post-stimulation (Kopp et al., 2010). N2 can be divided into subcomponents known as N2a, N2b, and N2c (Pritchard et al., 1991). Early studies employing the go/no-go task paradigm reported that N2 is more pronounced in the no-go condition than in the go condition. This finding suggests that N2 is associated with inhibition and error-monitoring processes. Additionally, under more challenging task conditions that require greater cognitive control, the amplitude of N2 is increased (Folstein and Van Petten, 2008).

The N1 component represents the initial stage of the attentional allocation process (Chacko et al., 2020) and peaks between 130 and 200 ms following stimulus onset. It is associated with visual stimulus discrimination and selective attention processing (Chacko et al., 2020). The N1 component has been found to originate from extrastriate and various posterior parietal areas (Di Russo et al., 2002).

The CNV was discovered in 1964 and is recognized as the first component identified in contemporary ERP research (Walter et al., 1964). The CNV is a stable, slow, negative waveform that is typically observed as peaks in the central and prefrontal regions. Negative potential deflections occur between warning stimuli and command stimuli, as well as during the presentations of warning stimuli, command stimuli, and the execution of commands. The first negative phase reflects the processing of the warning stimulus, while the second negative phase represents the preparatory potential associated with the individual’s readiness to respond to the target stimulus (Falkenstein et al., 2003).

## Characteristics of included studies

**Figure 1:**
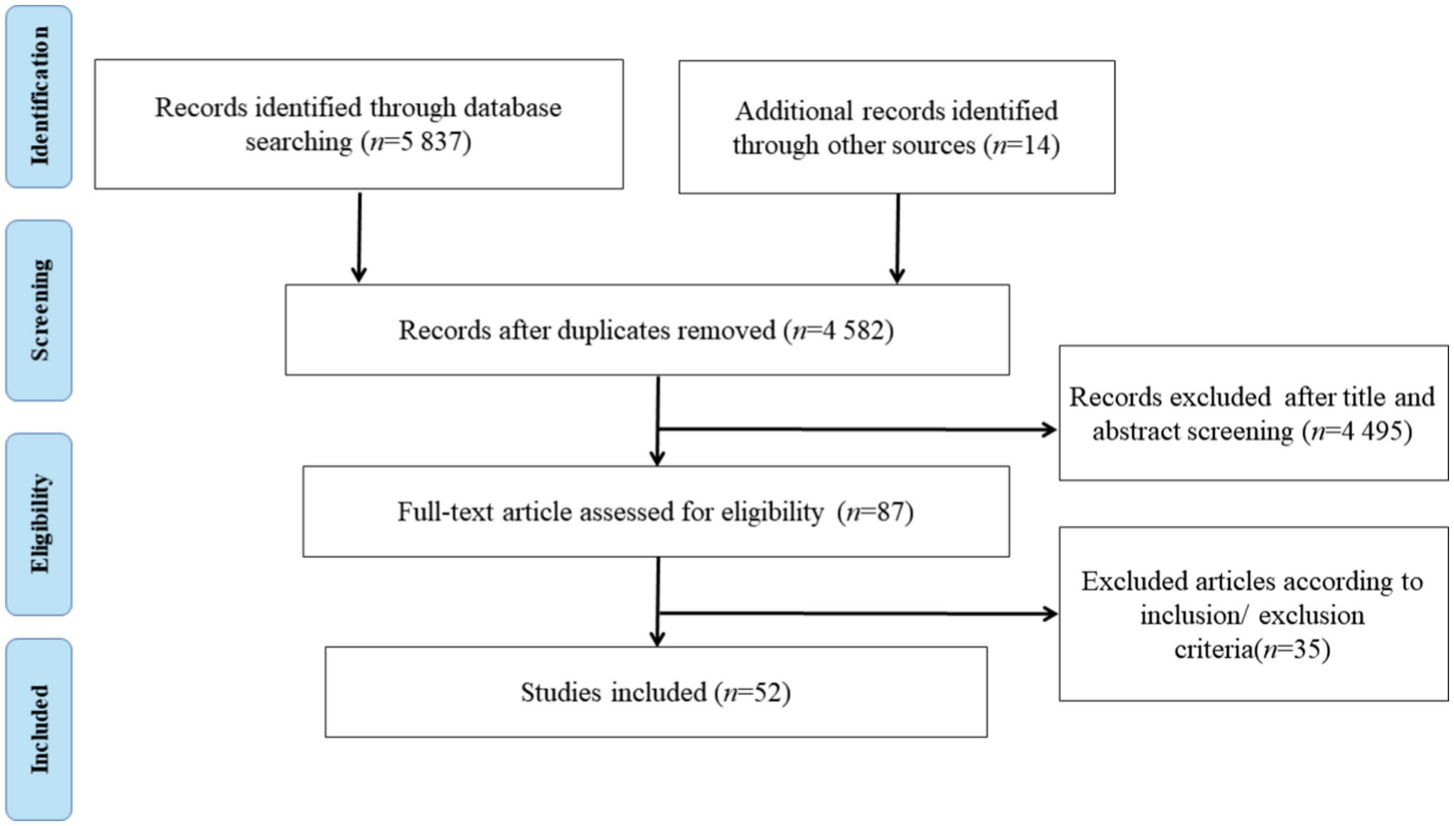
PRISMA study flow diagram of study selection.

A total of 5,837 relevant articles were retrieved during the search process. After removing 1,255 duplicates, 4,582 articles were retained following title and abstract screening. Subsequently, 87 full-text articles were reviewed, leading to the inclusion of 52 studies in the final review (Figure 1). Of these, 45 articles investigated inhibition, 3 articles examined working memory, and 4 articles explored cognitive flexibility (Table 3). Most studies employed a within-subjects crossover design, whereas only a few utilized randomized controlled designs. A total of nine ERP components were investigated, with the most frequently observed being P3 (43 studies), N2 (17 studies), and N1 (5 studies). Thirty-seven studies (86%) indicated that exercise increased P3 wave amplitude, whereas 13 studies (27.1%) reported shorter latency. Eight studies (53.3%) demonstrated an increase in N2 wave amplitude following exercise, whereas two studies (13.3%) observed shorter latency. Furthermore, two studies (40%) reported an increase in N1 amplitude after exercise, while another two studies (40%) found no significant change, and one study observed a decrease in amplitude. The remaining ERP components were reported less frequently, with each occurring in fewer than three studies.

**Table 3:** Detailed risk of bias quality assessment of each criterion for all studies included (*n* = 52).

Study ID	C1	C2	C3	C4	C5	C6	C7	C8	Total
Aly (2020)	1	0	1	0	1	1	1	1	6
Akatsuka et al. (2015)	1	1	1	0	1	1	1	1	7
Bae and Masaki (2019)	1	1	1	0	1	1	1	0	6
Bailey et al. (2021)	1	1	1	0	1	1	1	1	7
Chacko et al. (2020)	1	1	1	0	1	1	1	1	7
Chang et al. (2017)	1	0	1	0	1	1	1	0	5
Chu et al. (2015)	1	1	1	0	1	1	1	1	7
Chu et al. (2017)	1	1	1	0	1	1	1	0	6
Dimitrova et al. (2017)	1	1	1	0	1	1	1	1	7
Drollette et al. (2014)	1	1	1	0	1	1	1	1	7
Du Rietz et al. (2019)	1	1	1	0	1	1	1	1	7
Hillman et al. (2003)	1	1	1	0	1	1	1	0	6
Hillman et al. (2009)	1	1	1	0	1	1	1	0	6
Hsieh et al. (2018)	1	1	1	0	1	1	1	1	7
Hung et al. (2016)	1	1	1	0	1	1	1	0	6
Kamijo et al. (2004a)	1	1	1	0	1	1	1	1	7
Kamijo et al. (2007)	1	1	1	0	1	1	1	1	7
Kamijo et al. (2009)	1	1	1	0	1	1	1	1	7
Kao et al. (2017)	1	1	1	0	1	1	1	1	7
Kao et al. (2020a,b)	1	1	1	0	1	1	1	1	7
Kao et al. (2018)	1	1	1	0	1	1	1	1	7
Ligeza et al. (2018)	1	1	1	0	1	1	1	1	7
Lind et al. (2019)	1	0	1	0	1	1	1	1	6
Ludyga et al. (2017)	1	1	1	0	1	1	1	1	7
McGowan et al. (2019)	1	1	1	0	1	1	1	1	7
O'Leary et al. (2011)	1	1	1	0	1	1	1	0	6
Pontifex et al. (2013)	1	1	1	0	1	1	1	0	6
Pontifex and Hillman (2007)	1	1	1	0	1	1	1	1	7
Pontifex et al. (2021)	1	1	1	1	1	1	1	1	8
Scudder et al. (2012)	1	1	1	0	1	1	1	0	6
Stroth et al. (2009)	1	1	1	0	1	1	1	1	7
Swatridge et al. (2017)	1	1	1	0	1	1	1	1	7
Themanson and Hillman (2006)	1	1	1	0	1	1	1	1	7
Torbeyns et al. (2016)	1	1	1	0	1	1	1	1	7
Tsai et al. (2018)	1	0	1	0	1	1	0	0	4
Tsai et al. (2014)	1	0	1	0	1	1	1	0	5
Tsai et al. (2016)	0	0	1	0	1	1	0	0	3
Tsai et al. (2021)	1	1	1	0	1	1	1	0	6
Vonk et al. (2019)	1	1	1	0	1	1	1	1	7
Yu et al. (2022)	1	1	1	0	1	1	1	1	7
Wang et al. (2016)	1	1	1	0	1	1	1	0	6
Wang et al. (2015)	1	1	1	0	1	1	1	0	6
Wu et al. (2019)	1	1	1	0	1	1	1	0	6
Winneke et al. (2019)	1	1	1	0	1	1	0	1	6
Xie et al. (2020)	1	1	1	0	1	1	1	0	6
Li, G. G. (2020)	1	1	1	0	1	1	1	1	7
Li et al. (2020)	1	1	1	0	1	1	1	1	7
Wang and Zhou (2014)	1	1	1	0	1	1	1	1	7
Wen and Tsai, 2020	1	0	1	0	1	1	1	0	5
Won et al. (2019)	1	1	1	0	1	1	1	0	6
Zhou and Qin (2019)	1	1	1	0	1	1	1	1	7
Zhu and Zhou (2016)	1	1	1	0	1	1	1	1	7

C1 refers to random allocation; C2 refers to concealed allocation; C3 refers to baseline comparability; C4 refers to blinded assessors; C5 refers to between-group analysis; C6 refers to outcome point estimates; C7 refers to adequate assessment/reporting of exercise intervention; and C8 refers to adequate assessment/reporting of ERP component recording and data analysis.

The quality of the studies was assessed, revealing a generally low risk of bias, with except for blinded assessors. The majority (96%) of the studies included in this systematic review were classified as high quality according to the PEDro checklist, indicating a low risk of bias in the findings.

**Figure 2:**
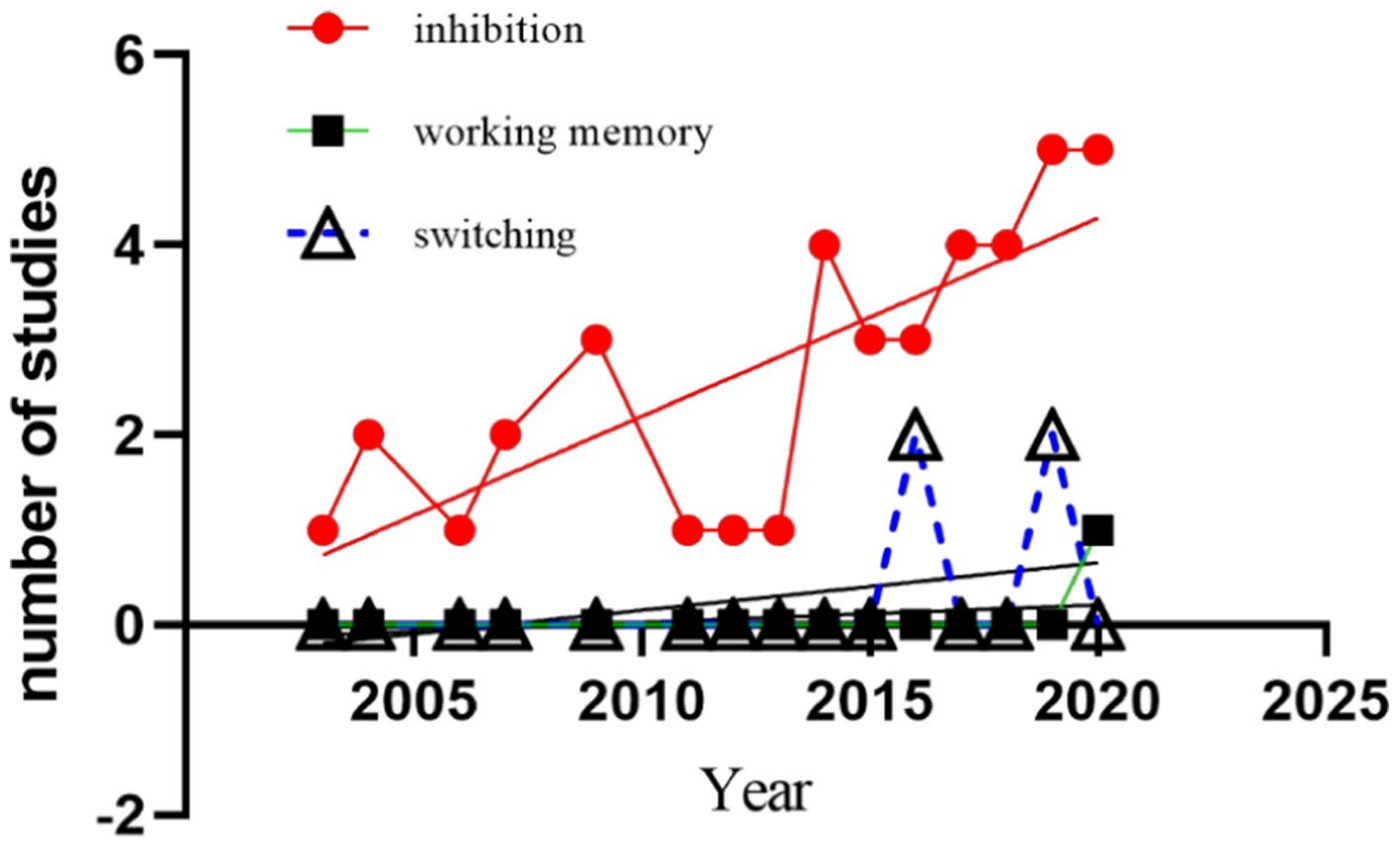
Number of published ERP studies of acute exercise on executive function.

As shown in Figure 2, the publication years of the included literature range from 2003 to 2023, with an average annual publication volume of 2.3. This indicates that research on the effects of acute exercise on cognitive function using ERP techniques remains in its early stages. Over the past decade, there has been an exponential increase in research on the effects of acute exercise on cognitive function, with 60% of these studies concentrating on executive function (Pontifex et al., 2019). However, there are relatively fewer studies employing ERP techniques to investigate the impact of acute exercise on executive function.

## Effects of acute exercise on executive function as measured by event-related potentials

### Timing of cognitive testing

Acute exercise refers to a single session of physical activity (Cai et al., 2020). Research on the effects of acute exercise on executive function has gained increasing attention in recent years. Recent experimental studies (Chang et al., 2019; Cui et al., 2020; McSween et al., 2021) and meta-analyses (Chen et al., 2020; Pontifex et al., 2019) indicate that acute exercise positively impacts executive function. The effects of acute exercise on cognitive function are transient, making it critical to explore the duration of these effects. Based on the chronological sequence of exercise and cognitive testing, cognitive tests are categorized into three phases: during exercise, immediate post-exercise, and delayed post-exercise. Immediate post-exercise refers to ERP testing conducted within 15 min after exercise, while delayed post-exercise refers to testing performed 20 to 55 min post-exercise. It has been concluded that 15 min post-exercise is the optimal time window for ERP testing (Chang et al., 2012).

Based on Chang’s categorization, 24 (46.2%) of the included studies conducted cognitive tests immediately following exercise, examining ERP components, including P3, N2, N1, P2, N140, N450 and CNV. All of these studies reported an positive effect on at least one ERP component. For example, Chang et al. (2015) found that 20 min of moderate-intensity cycling resulted in an increased P3 amplitude, a decreased N450 amplitude, and a shortened N450 latency post-exercise, while the N1 and N2 components remained unaffected. Xie et al. (2020) conducted 20 min of high-intensity interval cycling with young obese individuals and found that the amplitude of the late positive potential (LPP) increased, whereas the P3 amplitude remained unchanged.

**Figure 3:**
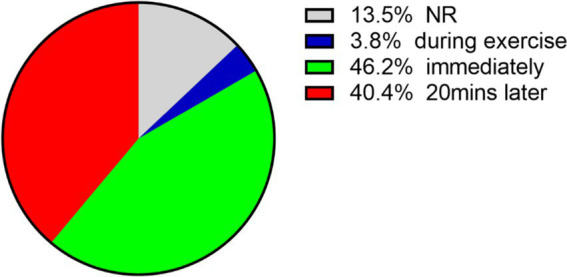
Time of cognitive test in the included literature. Note: Two studies included multiple testing times.

Twenty-one (40.4%) studies assessed cognitive tasks 20 min post-exercise (Figure 3). Only Themanson’s study observed that individuals with higher fitness exhibited larger error-related negativity (ERN) and P300 (Pe) amplitudes; however, it found no relationship between changes in ERP components and acute exercise. The remaining literature indicated that acute exercise improved executive function and ERP components across the corresponding paradigms to varying degrees.

Two (3.8%) studies tested ERP during exercise with inconsistent findings. Pontifex and Hillman (2007) investigated ERP during moderate-intensity cycling (60% of maximal heart rate) and observed a decrease in N1 and N2 amplitudes in parietal regions, accompanied by an increase in P2 and P3 amplitudes in the frontal lobes. This suggests that the allocation of attentional resources to gross body movements during exercise may be linked to inefficiencies in neural resource allocation, potentially leading to reduced interference control. In contrast, Torbeyns et al. (2016) employed a power bicycle at a self-selected speed for 30 min and administered the Stroop task concurrently with exercise. The study found no difference in accuracy under exercise conditions; however, N2 amplitude and P3 latency were notably affected, demonstrating positive effects on inhibitory function. The discrepancies between the studies may be attributed to several factors: Torbeyns included sedentary individuals engaging in moderate to high-intensity exercise for an average of 2.5 h per week, whereas Pontifex included university students with no restrictions on their exercise habits. The intensity of the exercises varied, with Pontifex employing 60% of maximal heart rate and Torbeyns utilizing a self-selected speed. Relatively low exercise intensity may be insufficient to impair executive function in individuals with regular exercise habits. According to a meta-analysis by Lambourne and Tomporowski (2010), cognitive function tends to be impaired during exercise but shows improvement afterward. The transient frontal function decline theory posits that strong activation of motor and sensory systems during exercise may compromise higher cognitive functions in the prefrontal cortex (Dietrich, 2003). During cognitive tasks performed concurrently with exercise, both the exercise and cognitive tasks compete for limited cerebral blood oxygen and glucose, which may temporarily interfere with frontal lobe function and result in diminished performance on executive function tasks.

In summary, test timing is a critical factor influencing ERP outcomes. Our findings suggest that the effect of exercise on enhancing executive function may last for approximately 40 min; however, the optimal measurement time remains to be determined. The mechanism underlying the immediate effects of acute exercise on cognitive function may be associated with changes in physiological arousal. According to the Yerkes-Dodson inverted U-shaped model, an optimal level of arousal enhances cognitive performance (Yerkes and Dodson, 1908). The arousal level induced by acute exercise is temporary and gradually diminishes post-exercise, returning to baseline levels approximately 20 min after cessation of exercise. The literature reviewed generally supports this principle. The choice of ERP components can significantly influence the observed effects of acute exercise interventions, as these components reflect distinct cognitive processes. Similarly, variations in electrode placement can lead to inconsistent recordings of neural activity, potentially obscuring the true effects of exercise.

### Different subdomains of executive function

Differences may exist in the effects of acute exercise interventions on the various subdomains of executive function. Some studies have reported significantly greater effects of acute exercise on inhibition compared to working memory and cognitive flexibility (Chen et al., 2011), whereas others have observed more substantial intervention effects on working memory than on cognitive flexibility and inhibition (Weng et al., 2015; Cai et al., 2020). Conversely, other research has found no significant differences in the effects across these three subdomains (Verburgh et al., 2014).

**Figure 4:**
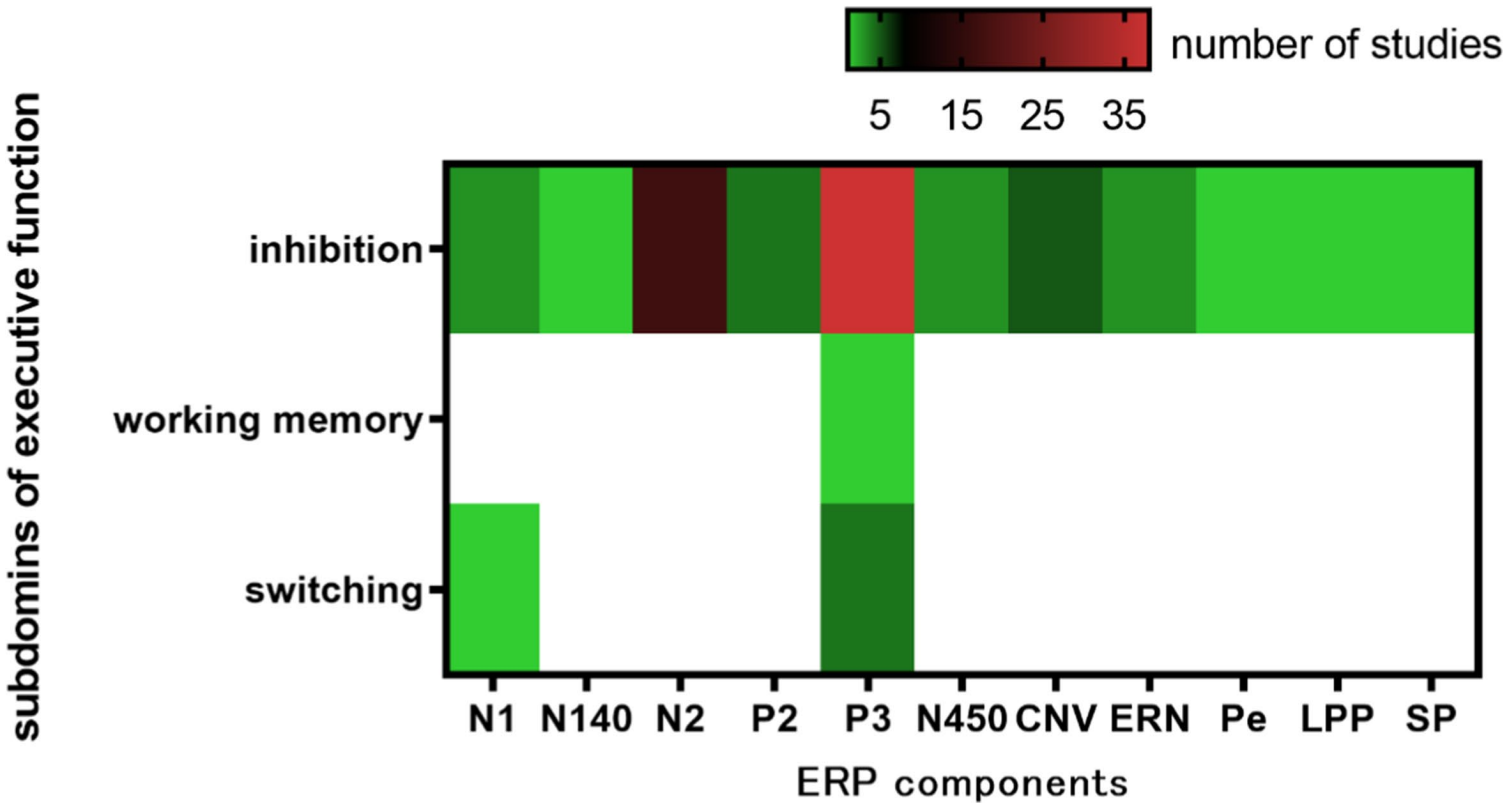
Heatmap of executive function and ERP components.

Inhibition represents a crucial component of executive function. Pontifex et al. (2019) found that 60% of the literature on acute exercise interventions targeting cognitive function focused on executive function, with 41% specifically examining the effects of inhibition. The results of the present study align with previous findings, as 46 of the included papers (Figure 4) investigated inhibition (88.5%), with the primary ERP components being P3, N2, and CNV, which exhibited increased amplitudes and shortened latencies. Test paradigms for inhibition, such as the Flanker, Go/No-Go, and Stroop tasks, demonstrate that acute exercise enhances attentional resources, aids in the classification and evaluation of executive function stimuli, and improves both conflict inhibition and response inhibition.

Only two studies in the included literature utilized acute aerobic exercise as an intervention for working memory. Both studies employed a within-subject design to assess N-back and AX-CPT performance, as well as ERP components, in college students under both resting and moderate-intensity aerobic exercise conditions. The results indicated a significant increase in P3 amplitude following aerobic exercise, whereas P3 latency, as well as N2 amplitude and latency, did not exhibit significant changes (Kao et al., 2020a,b; Scudder et al., 2012). These findings suggest that aerobic exercise may enhance goal maintenance processes and more effectively inhibit external neural activity, thereby allocating additional attentional resources for goal updating and modification. Three studies in the included literature examined the switching function and found that switching costs were significantly lower in the exercise condition compared to the control condition, with a larger P3 amplitude observed (Bae and Masaki, 2019; Hung et al., 2016; Wu et al., 2019).

As noted in the review, ERP research on acute exercise has primarily focused on inhibition, yielding relatively consistent results. However, there are comparatively fewer studies on working memory and cognitive flexibility, and the findings in these domains have been inconsistent. The predominance of inhibition research stems from its methodological maturity, urgent clinical demands, and early theoretical advancements, whereas the inherent complexity of working memory and cognitive flexibility, coupled with measurement challenges and indirect application value, have contributed to relative research stagnation.

### Different participants

The literature predominantly focused on young individuals, comprising 39 studies, including two on drug addiction, one on obesity, and one on criminal behavior. Five studies were conducted on older adults, including one on mild cognitive impairment, while three addressed both older and younger populations. Additionally, five studies involved children and adolescents, with three specifically focusing on children diagnosed with attention-deficit/hyperactivity disorder (ADHD). Overall, the research subjects predominantly consist of young individuals, with fewer studies involving older adults, children, and adolescents. Recent research has increasingly focused on specific populations, including those with ADHD, addiction issues, criminal behavior, and obesity.

Age may act as a moderating factor influencing the relationship between acute exercise and ERP components. Ludyga et al. (2016) meta-analysis concluded that older adults and children exhibited significantly greater enhancements in executive function following acute exercise compared to adolescents and adults. Hillman et al. (2009) study found that 20 min of running at 60% of maximal heart rate significantly improved inhibition in children, as evidenced by higher accuracy on the Flanker task and increased P3 amplitude in the exercise condition. Drollette et al. (2014) categorized 40 children into high- and low-inhibition groups based on their Flanker task performance and had them engage in 20 min of jogging at 60–70% of maximal heart rate. Both groups exhibited shorter P3 latencies and lower N2 amplitudes compared to the sedentary condition, indicating that acute exercise enhances efficiency in stimulus categorization and reduces response conflict. However, P3 amplitude increased in the low-inhibition group, whereas no significant change was observed in the high-inhibition group. This study is the first to report the effects of acute exercise on N2 components, revealing that a decrease in N2 amplitude indicates enhanced brain efficiency and reduced conflict. This implies that children with lower inhibition experienced greater benefits from acute exercise. Similar results were observed using fMRI, indicating significant increases in frontal and parietal activation and reductions in reaction time before and after exercise (Chen et al., 2011). Pontifex et al. (2013) reported a 4% increase in Flanker task accuracy, a 38% increase in P3 amplitude, and a decrease in P3 latency following 20 min of running at 65–75% of maximal heart rate in individuals with ADHD compared to the control condition.

Kamijo et al. (2007) discovered that moderate-intensity aerobic exercise (50% maximal oxygen uptake) increased P3 amplitude in young adults, while both low-intensity (30% maximal oxygen uptake) and moderate-intensity exercises resulted in reduced P3 latency in both older and younger adults. This indicates that low-intensity acute exercise may also confer benefits for executive function in older adults. Similarly, Dimitrova et al. (2017) found that 20 min of moderate-intensity exercise (60% of reserve heart rate) improved Stroop task performance in both young and elderly individuals. Notably, the amplitude of the ERP component associated with incongruent tasks increased more significantly in elderly participants.

In summary, the current study indicates that acute exercise significantly impacts both the latency and amplitude of ERP components in younger individuals, while older individuals show a greater sensitivity to changes in latency alone. This discrepancy may be attributed to electrophysiological variations across different age groups.

### Exercise prescription

The results indicated that 42 out of 52 studies focused on inhibitory control, with moderate-intensity aerobic exercise lasting between 16 and 35 min demonstrating a positive effect on P3 and N2 components. The review suggests that current ERP research methods are somewhat limited and recommends incorporating advanced techniques, such as source localization and frequency-domain analysis. Furthermore, it highlights the need to investigate working memory, cognitive flexibility, and other executive functions in greater depth, as well as to further explore the “dose” effect of exercise through additional experimental studies. Understanding how the dose of exercise affects executive function is crucial. Previous meta-analyses and systematic reviews have investigated the impact of exercise dose on the improvement of executive function (Chang et al., 2012; Cai et al., 2020). To further elucidate the relationship between physical exercise doses and ERP components, the American College of Sports Medicine (2018) “Guidelines for Exercise Testing and Prescription” (10th edition) were utilized to categorize exercise intensity and type (American College of Sports Medicine, 2018). Physical exercise types are categorized as aerobic, resistance, flexibility, and balance exercises.

**Figure 5:**
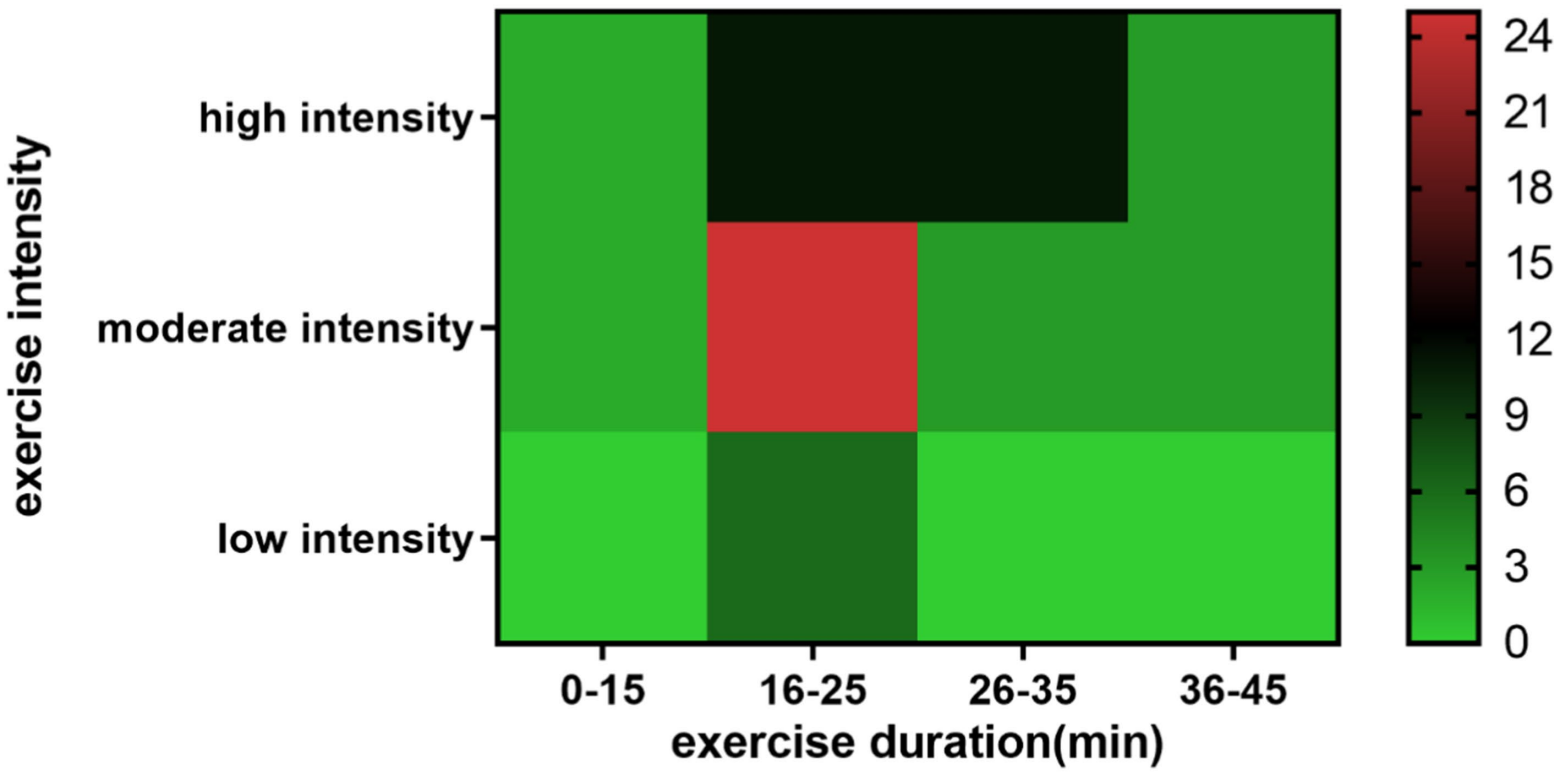
Heatmap of exercise intensity and duration.

For ease of interpretation, the frequency of physical activity doses is represented by a heat map (Figure 5), which depicts the relationship between exercise intensity and duration. Moderate intensity was the most frequently reported in the included literature (71.7%), followed by high intensity (58.7%); low intensity was the least used (13%) (11 studies contained more than 2 experimental groups). These findings align with the inverted U-shaped model, which is supported by numerous studies. Kamijo et al. (2004b) found that moderate-intensity exercise increased CNV and P3 amplitude, indicating that acute exercise induces optimal arousal and enhances attentional resource allocation to executive function tasks. However, both low- and moderate-intensity exercises were found to shorten P3 latency in young and older adults alike (Kamijo and Takeda, 2009). Chang et al. (2017) observed that moderate-intensity exercise increased P3 amplitude in the context of the Stroop task and shortened N450 latency. Ligeza et al. (2018) reported that moderate-intensity exercise increased N2 amplitude in the Flanker task relative to both sedentary conditions and high-intensity interval exercise. It has also been suggested that the inverted U-shaped model may vary depending on the type of cognitive task, with tasks of lower complexity potentially benefiting more from high-intensity physical activity (Mcmorris et al., 2016). Chang and Etnier (2009) suggested that high-intensity resistance exercise enhanced information processing, whereas moderate-intensity resistance exercise more effectively enhanced aspects of cognitive control. High-intensity exercise has a transient negative impact on prefrontal-dominated executive function by elevating blood lactate levels, and is more sensitive to young people and has less impact on the elderly (Coco et al., 2020). In addition, individual differences (such as those with physically active individuals) can also affect the improvement of exercise on ERP components and cognitive function (Massimino et al., 2023).

Exercise duration and intensity are intrinsically interconnected indicators of physical activity. Exercise intensity is inversely related to duration; as intensity increases, the maximum duration achievable decreases. Acute exercise-induced changes in executive function may require a minimum exercise duration. The literature predominantly employs durations of 16–35 min (94.3%), while durations of 15 min or less, as well as 36–45 min, are less common, comprising only about 5% of studies. Chang et al. (2012) found that a minimum of 11 min of exercise was required to produce changes in cognitive function, with no improvement observed for exercises lasting 10 min or less. A previous study by our team found that neither exercise lasting less than 10 min nor that lasting more than 20 min improved executive function in middle-aged and older adults; only exercise lasting between 11 and 20 min significantly enhanced executive function (Cai et al., 2020). In this study, we observed that acute exercise lasting between 16 and 25 min was associated with an increase in P3 amplitude, whereas only one instance of exercise lasting 26–35 min showed a decrease in P3 amplitude. This suggests that the exercise durations utilized in the included studies are appropriate. However, it should be noted that 10 and 20 min merely represent key time thresholds, reflecting the characteristics of the included studies, and only offer a general estimate of the effect of exercise duration. The optimal exercise duration necessitates further investigation through additional experiments.

Aerobic exercise predominated in the included literature, with cycling and running being the most commonly utilized forms, while soccer, video games, Tai Chi, and resistance exercises were employed less frequently. Tsai and Wang (2015) found that open-skill exercises resulted in smaller switching costs, reduced reaction times, and increased P3 amplitudes compared to those of closed-skill and control groups. Tsai et al. (2018) reported that both aerobic and resistance exercises improved behavioral performance and increased P3 amplitudes in older adult. O'Leary et al. (2011) found that aerobic exercise increased P3 amplitude; however, sports games and electronic games did not demonstrate similar benefits.

In summary, most studies primarily utilized aerobic exercises, such as running and cycling, while other types of exercise were used to a lesser extent. Variations in exercise modalities may engender divergent results, given their differential engagement of distinct physiological systems and cognitive mechanisms. Moderate-intensity exercise, lasting between 16 and 25 min, was found to improve ERP components, such as P3 and N2.

### Mediation of cardiovascular fitness

The level of cardiovascular fitness may influence the relationship between acute exercise and ERP components, with the “cardiovascular function hypothesis” serving as a classical mechanism to explain how physical activity enhances cognitive function (Etnier et al., 2006). Many studies have identified cardiovascular fitness as a crucial variable affecting cognitive function within the framework of this hypothesis. Tsai et al. (2016) categorized participants into two groups based on their cardiovascular fitness levels and conducted a 30-min run at 60% of maximal oxygen uptake. The study found that only individuals with higher cardiovascular fitness experienced smaller switching costs and larger P3 amplitudes following acute exercise. This supports the notion that the neural mechanisms underlying the effects of acute exercise may depend on an individual’s level of cardiovascular fitness. Stroth et al. (2009) found that after 20 min of running at 60% of maximal oxygen uptake, adolescents with high cardiovascular fitness exhibited larger CNV amplitudes and smaller N2 amplitudes, whereas P3 amplitude was not significantly associated with either acute exercise or cardiovascular fitness. Themanson and Hillman (2006) demonstrated that after 30 min of running at 60% of maximal heart rate, young individuals with high cardiovascular fitness exhibited larger ERN amplitudes, while Pe amplitudes were not significantly associated with acute exercise. This suggests that cardiovascular fitness may influence executive function performance and electrophysiological indices more than acute exercise alone. Improved cardiovascular fitness may lead to increased cerebral blood flow and elevated levels of brain-derived neurotrophic factors, which in turn manifest as changes in both the amplitude and latency of ERP components. Therefore, cardiovascular fitness should be considered an important variable in study design.

## Limitations and conclusion

This paper examines the effects of acute exercise on ERP components associated with executive function tasks. ERP, which reflects brain neuroelectric activity occurring between stimulus presentation and response, offers a unique approach for elucidating how acute exercise enhances executive function. Combining ERP with behavioral performance improves the understanding of the mechanisms underlying the relationship between physical activity and cognition. However, this review has several limitations: (1) language bias (English/Chinese studies only); (2) heterogeneity in exercise protocols limiting dose–response analysis; and (3) insufficient data synthesis methods for ERP components.

The present study found that acute exercise has a positive impact on executive function and ERP components, with benefits becoming evident not during exercise but rather during the period following exercise cessation. However, the optimal measurement window may not be strictly limited to 15 min, and a broader time frame could be more effective. Therefore, investigating the duration of cognitive benefits derived from exercise should be a focus of future research. Most studies utilized aerobic exercises, such as cycling and running, while fewer studies explored resistance exercise, Tai Chi, and football. Future research should encourage a diverse range of physical activities to meet the exercise needs of various populations. This approach may enhance physical fitness levels and elevate cognitive demands. Regarding exercise dosage, most studies employed moderate-intensity exercise, while others incorporated high-intensity interval training. Given the significant variation in exercise intensity, there is currently no consensus on the optimal intensity for physical activity. This issue necessitates resolution through enhanced experimental design.

Future studies are encouraged to adopt more diverse interventions for different populations, such as the dual-task protocol of exercise combined with cognitive training. Exercise may improve cognition by releasing the brain-derived neurotrophic factors, while cognitive training can directly enhance executive functions. It seems that the association of active exercise and cognitive training in a dual task protocol could enhance the improvement on executive functions (Deodato et al., 2024).

## Data Availability

The original contributions presented in the study are included in the article/supplementary material, further inquiries can be directed to the corresponding author.

## Glossary

ADHD: attention-deficit/hyperactivity disorder
AE: aerobic exercise
AR: Accuracy rate
C: control group
CNV: Contingent Negative Variation
CPT-OX: cued continuous performance task
D: duration
EI_H_: higher fitness group
ERN: error-related negativity
ERP: event-related potentials
fMRI: functional magnetic resonance imaging
HIIE: high intensity interval exercise
HR: heart rate
HRmax: maximum heart rate
HRR: heart rate reserve
I: intensity
LPP: late positive potential
MCI: mild cognitive impairment
MA: methamphetamine addict
MICE: moderate intensity continuous exercise
N: sample size
NR: no report
PRISMA: Preferred Reporting Items for Systematic Reviews and Meta-Analyses
RE: resistance exercise
RF: resting
RM: repetition maximum
RPE: rating of perceived exertion
RT: reaction time
SP: sustained potential
SRF: small-sided real football game
SWF: small-sided walking football games
VO2max: maximum oxygen consumption
